# Plant and marine-derived natural products: sustainable pathways for future drug discovery and therapeutic development

**DOI:** 10.3389/fphar.2024.1497668

**Published:** 2025-01-06

**Authors:** Muhammad Ahmad, Maleha Tahir, Zibin Hong, Muhammad Anjum Zia, Hamza Rafeeq, Muhammad Shaheez Ahmad, Saif ur Rehman, Junming Sun

**Affiliations:** ^1^ Guangdong Cardiovascular Institute, Guangdong Provincial People’s Hospital-Ganzhou Hospital, Guangdong Academy of Medical Sciences, Guangzhou, China; ^2^ Institute of Physiology and Pharmacology, Faculty of Veterinary Science, University of Agriculture Faisalabad, Faisalabad, Pakistan; ^3^ Enzyme Biotechnology Lab, Department of Biochemistry, University of Agriculture Faisalabad, Faisalabad, Pakistan; ^4^ Laboratory Animal Center, Guangxi Medical University, Nanning, Guangxi, China

**Keywords:** bioactive compounds, bioprospecting, biosynthetic pathways, metabolites, gene cluster, antioxidants, microbes, sustainable practice

## Abstract

Plant- and marine-derived natural products are rich sources of bioactive compounds essential for drug discovery. These compounds contain complex mixtures of metabolites, which collectively contribute to their pharmacological properties. However, challenges arise in the isolation of individual bioactive compounds, owing to their intricate chemistry and low abundance in natural extracts. Despite these limitations, numerous plant and marine-derived compounds have achieved regulatory approval, particularly for treating cancer and infectious diseases. This review explores the therapeutic potential of plant and marine sources along with innovative extraction and isolation methods that support sustainable drug development. Future perspectives will highlight the role of responsible innovation, artificial intelligence, and machine learning in advancing drug discovery, underscoring the importance of continued research to meet global health needs.

## Introduction

Natural products (NPs) are integral to medicinal practices. Currently, the potential of plant- and marine-derived compounds in sustainable drug discovery is receiving renewed attention, especially in addressing challenges, such as antibiotic resistance and cancer treatment (Hassan ul et al., 2022b). According to the 1985 World Health Organization (WHO), almost 65% of the global population receives primary healthcare from plant-derived medicines, with a lower incidence in developed countries (Alves et al., 2018; Atanasov et al., 2021). NPs, with their structurally complex and biologically pre-validated arrangements, interact efficiently with specific targets (Newman and Cragg, 2007). Nanoparticles play a significant role in drug development, particularly in the discovery of pharmaceuticals. As of 2005, nanoparticles comprised approximately 40% of all pharmaceuticals (Butler, 2005). In December 2004, the FDA approved the first marine-derived compound, ziconotide intrathecal infusion (Prialt), for the treatment of severe pain in the United States. In October 2007, the European Union appmany roved trabectedin (Yondelis, PharmaMaritss the first marine anticancer drug (Newman and Cragg, 2014).

NPs from plants, animals, and minerals have long been used to treat human diseases (Radjasa et al., 2011). NPs research continues to focus on various structures that can be used as templates in the pharmaceutical industry to develop new drugs (Rangel and Falkenberg, 2015; Haroon Ur Rashid et al., 2024). These approved substances represent a wide range of chemical diversities, demonstrating the importance of natural compounds in current drug discovery efforts. Since the first isolation of an NP in its pure form (i.e., morphine) from opium by Serthürner in 1805, NPs have played a key role in drug discovery and development. NPs have long been valued for their potential in drug discovery and development, and their importance has grown with advancements in technology and scientific understanding (Butler, 2008; Gray et al., 2012; Turnaturi et al., 2023).

In the early 1950s, the first marine NPs, arabino-nucleosides pongothymidine and spongouridine, were isolated from extracts of the sponge *Tectitethya crypta*, which has a clear impact on the development of drugs for human use (Paz-Ares et al., 2017; Varijakzhan et al., 2021). It remains unknown whether they are produced by sponges or by other microorganisms attached to the sponges. However, other nucleosides found in *T. crypta* extracts are of bacterial origin (Bertin et al., 2015; Barreca et al., 2020). Spongothymidine and spongouridine greatly enhanced the generation of nucleosides and eventually resulted in cytarabine (ara-C, arabinosylcytosine) production as an anti-leukemic drug and vidarabine as an antiviral agent (ara-A, arabinosyladenine) (Zöllinger et al., 2007). This review aims to explore the role of plant- and marine-derived natural products in modern drug discovery, focusing on their potential to treat life-threatening diseases such as cancer, cardiovascular disorders, and infectious diseases. Furthermore, we discuss innovative extraction techniques and challenges associated with scaling up natural compounds for pharmaceutical applications.

### Drug development and discovery

NPs and their derivatives, known as secondary metabolites, have been used in the evolution of new medicinal drugs since ancient times (Hamann et al., 2007; Muhammad et al., 2024). Although NPs have been used for centuries, recent technological advancements in genomics, bioinformatics, and extraction techniques have significantly expanded their applications in modern drug discovery. These innovations have enabled the isolation and characterization of bioactive compounds with unprecedented precision, thereby opening new avenues for the development of novel therapeutics. Prior to the 20th century, human and animal treatments have relied on raw or partially refined extracts from various sources. The advent of receptor theory in the 20th century transformed drug development, highlighting the importance of drug-chemical interactions with biological molecules, such as proteins, DNA, and RNA. This concept has guided scientists to target the chemical properties of NPs for therapeutic application (Pereira, 2019). Isolated NP compounds are the primary treatment strategy for different diseases in their pure forms, ushering in a new era of pharmacology. The chemical structures of other bioactive compounds responsible for the action of the drug extracts have been previously elucidated (Kobayashi, 2016). NP research continues to examine various types of structures developed in different pharmacies to manufacture new drugs (Li et al., 2015; Wu et al., 2024).

### Resources of natural products

Marine Natural Products (MNPs) are bioactive metabolites sourced from different marine organisms such as algae, cyanobacteria, sponges, dinoflagellates, mollusks, mangroves, fish, and soft corals. The marine environment is a living place for 34–35 known phyla in the animal kingdom, eight of which are exclusively aquatic (Fusetani and Kem, 2009). Between 1985 and 2012, many marine NPs with bioactivity of approximately 75% were isolated from invertebrates and cnidarians, and approximately 57% were bioactive NPs. These organisms lack defense mechanisms; instead, they release secondary metabolites such as toxins as a chemical defense strategy (Hu et al., 2015). In 2013, sponge-associated species produced 22 percent of the 411 NPs isolated from marine actinomycetes (Hernández-Ledesma and Herrero, 2013). Since 2008, approximately 1,000 or more NPs have been isolated, and by the end of 2015, the total number of isolated natural products reached approximately 27,000 (Fusetani and Kem, 2009).

### Role in drug development

**FIGURE 1:**
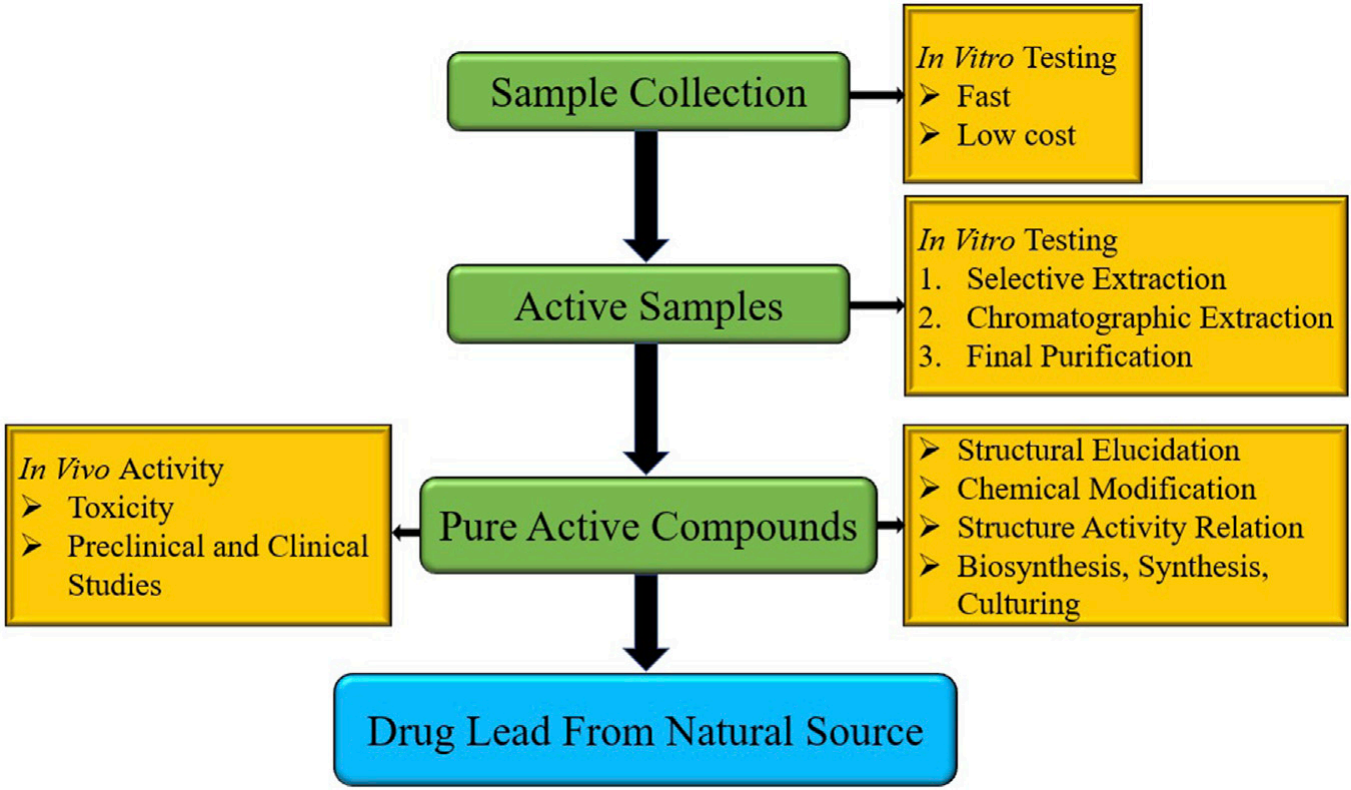
Discovery and Steps drug lead compounds from natural resources.

The discovery of NPs has attracted attention from the pharmaceutical industry. Natural products derived from plants and microbes have been used for the treatment of various diseases. This is crucial for the development of new compounds for drug development (Patra et al., 2018; Thomford et al., 2018). Natural-source drugs have also been used as drug leads, and can be improved using synthetic methods. The entire stepwise extraction procedure for the source and accumulation of bioactive compounds is shown in Figure 1 (Najmi et al., 2022; Yusuf et al., 2023).

**FIGURE 2:**
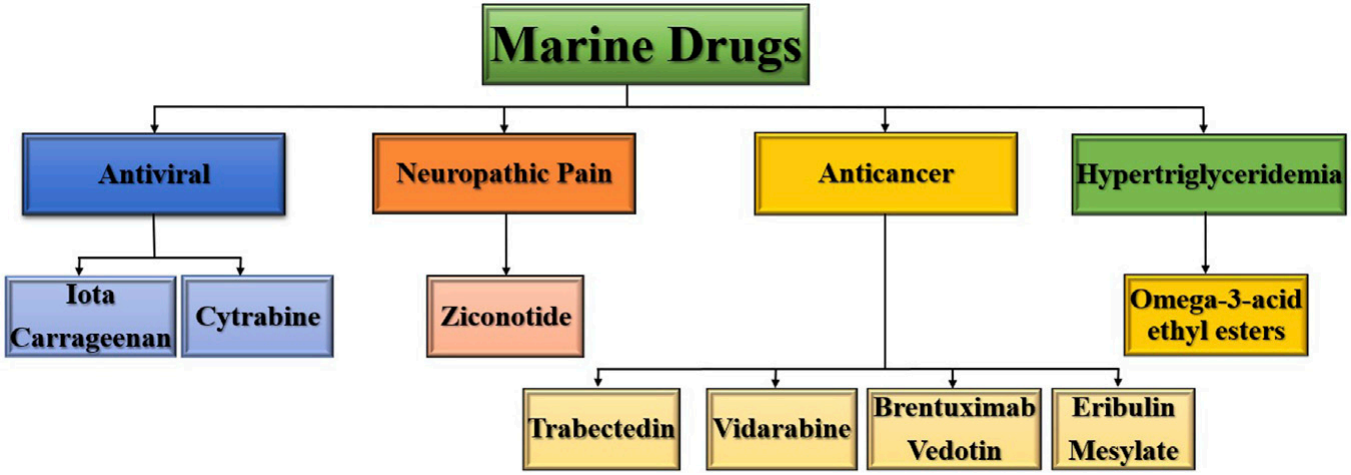
FDA or EMEA-approved marine drugs.

NPs are increasingly being studied in marine environments in addition to terrestrial organisms (Martins et al., 2014). Consequently, natural drugs discovered from marine sources have high efficacy and specificity in the treatment of a wide range of human diseases. Natural marine drug discovery has advanced significantly in recent decades, with at least eight related drugs approved by the US Food and Drug Administration and European Medicines Agency (EMEA) (Figure 2) (Zhang et al., 2016). Many plants were discovered to have medicinal properties in the early years and have been used to treat various pathological conditions. Compared with the same compounds from terrestrial sources, secondary metabolites from marine sources have shown that marine NPs have a higher incidence of significant bioactivity, which is frequently associated with a high degree of structural novelty (Cheung et al., 2015).

## Plant-based natural products (NPs)

The Plant kingdom accounts for a large proportion of NPs recorded in the Dictionary of Natural Products (DNP). This is consistent with the plants reported by Bérdy (2012), which account for approximately 70 percent of the NP. This is not unusual from a historical viewpoint because the first natural products to be investigated were medicinal plants. It began with German pharmacist Friedrich Sertuerner who isolated morphine (alkaloid) in 1817 (Chassagne et al., 2019). In the 19th century, it gained popularity by discovering a wide variety of natural plant compounds. A high percentage of terpenoids, which are prominent in gymnosperms and di-cotyledons, is the main characteristic of metabolites found in DNP. This class was regarded as the most significant and complex NP class. They have various roles in plant production, growth, coordination, and protection. Notably, they exhibited antineoplastic behavior. Limonene, Tanshinone, Celastrol, and Lycopene are terpenoids (Huang et al., 2012; Aljohani, 2023).

The most confined NPs of the Plant kingdom come through di-cotyledons (83,7 percent), followed by monocotyledons (8.1 percent), and Gymnesperms (3 percent). Liver worts, ferns, fern allies, and mosses, including the rhodophyte and chlorophyte groups, constitute only a small part of the NP reported. In addition, three botanical groups host approximately one-fourth of the total (Compositae, Leguminosae, and Labiatae) (Bennet, 2006; Foghis et al., 2023).

This Composite family is the largest global group of blooming plants, encompassing more than 32,700 species. The Leguminosae family is the third largest genus with over 20,800 species and more than 32,700 flowering plants globally (Roskov et al., 2014; Shah et al., 2020). Approximately half of the compounds in the Leguminosae family are flavonoids, with a diverse range including quercetin, kaempferol, and their derivatives (Russo et al., 2016). Since 2011, 44 products from this family have been licensed or clinically approved, making it the most prolific botanical group in terms of drug development (Zhu et al., 2011). The Labiatae family is also notable, ranking as the third largest botanical genus. Approximately 71% of the compounds isolated from this group were terpenoids. Various studies on volatile oils in economically important generations have clarified these findings (e.g., La vandula, Mentha, and Thymus). *Salvia* species account for about 20% of the total NPs derived from the Labiatae, the most representative being *Salvia miltiorrhiza* Bunge. *S. miltiorrhiza* (in Chinese Danshen) is a common traditional Chinese herb used to treat several cardiovascular diseases, hyperlipidemia, and cerebrovascular conditions (Wu et al., 2012). The major bioactive components in this genus are lipophilic diterpenoids (tanshinones) and hydrophilic phenolic acids (salvianolic acids). These substances have various pharmacological effects, including anticancer and cardiovascular effects (Hosseini and Ghorbani, 2015; Shaito et al., 2020). More than 30 clinical trials have been conducted on *S. miltiorrhiza* and its tanshinones to validate their application in stroke, angina, and other ischemic disease survivors. In addition, Dantonic, the Chinese botanic compound with *S. miltiorrhiza* extracts, is being treated for angina poctoris and cardiovascular disorder in phase III clinical studies (completed throughout 2016) by the US Food and Drug Administration (FDA) (Chao et al., 2017).

## Marine based natural products (NPs)

**FIGURE 3:**
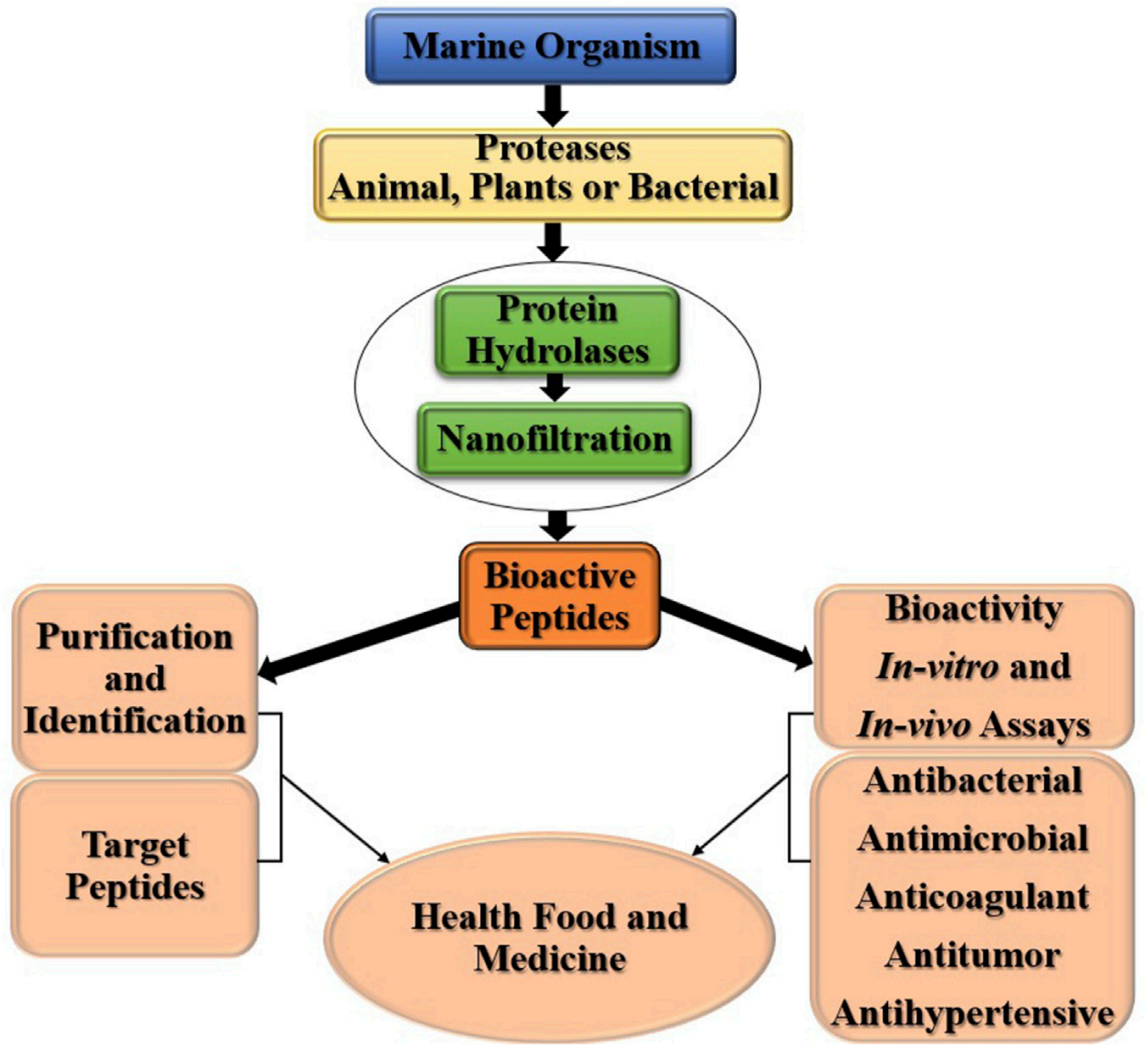
Drug Discovery from marine sources.

Marine organisms, including tunicates, mollusks, sponges, bryozoans, and many other cyanobacteria and bacteria are rich sources of inspiration for drug discovery. Marine environments provide a novel lead against various diseases caused by bacterial, fungal, viral, and parasitic infections, all of which may develop resistance to available drugs. Ecteinascidin-743, adenine, dolastatin 10, and kahalalide fares (KF) are commonly used in advanced clinical trials. Many marine natural products (MNPs) have been used in clinical trials to treat cancer, emerging infections, and other diseases that have developed drug resistance. The threat of bioterrorism to the treatment of infectious organisms has contributed to the assessment of natural ocean products (Figure 3), highlighting the steps involved in drug discovery from marine sources (Donia and Hamann, 2003).

NPs are a significant contributor to drug discovery reported in the literature (Newman and Cragg, 2016b). The chemical diversity of NP is ideally aligned with drugs, rather than with synthetic libraries, making them ideal candidates for drug discovery projects. Their commercialization hinged on the advancement of sample collection techniques as well as spectrometric, primarily NMR, and separation methods. By the end of 2016, approximately 28,500 MNPs had been identified. Most biological activities of MNPs have been attributed to their cytotoxic and anticancer properties (Newman and Cragg, 2016a).

Approximately 70% of the Earth is covered with water and is home to a diverse range of species, of which 34 of the 35 taxonomic animal phyla are inhabitants of these water bodies worldwide (Altmann, 2017). The first living organism to inhabit the marine environment was estimated to be present on Earth more than 3,500 million years ago. Marine environments are harsher than terrestrial environments, resulting in the production of a considerable variety of NPs by organisms that live in water. The functional and structural diversities of these marine-based compounds are enormous, and they have a higher incidence of significant bioactivities. More than 30,000 MNPs have been discovered since the first report of a biologically active MNP, spongothymidine, was published in 1950. However, this represents only a fraction of the potential scaffolds yet to be explored as less than 5% of the deep sea has been investigated (Romano et al., 2017).

### Marine based drugs

NPs play a significant role in drug development by enhancing technologies used by different drug-producing industries worldwide. Although synthetic compounds dominate the pharmaceutical market, a significant proportion of the approved drugs originate from natural sources. These MNP-derived pharmaceuticals account for approximately half of all the known medical products. As we continue to explore ocean depths, marine-based NPs remain a promising avenue for novel drug discovery (Stonik, 2009). In addition to their potential in novel drug discovery, marine-based NPs offer numerous opportunities for the development of new biomaterials and medical devices. Furthermore, marine-based NPs could provide a source of inspiration for the design and synthesis of advanced functional materials with unique properties such as self-healing capabilities and enhanced mechanical strength.

### Marine-derived anti-cancer drugs

The most severe human disease is cancer, which is becoming more common due to changes in cell maturation, nutrition, the environment, and lifestyle. Cancer treatments are ineffective and some side effects have been observed with currently available drugs (Ali et al., 2022; Ahmad et al., 2024). Approximately 80% of the population in developing countries primarily uses plant-based drugs (Aziz et al., 2018). Drugs of natural origin account for nearly 60% of the cancer treatments approved by the FDA. Vitamins B, C, and E, fibers, and carotenoids are abundant in vegetables and fruits, and these nutrients contribute to the cancer-fighting properties of food. There is a link between increased natural antioxidant intake in the diet and a lower risk of different diseases, such as cancer and coronary heart disease (Rios et al., 2009; Ul Hassan et al., 2021). Recent studies have shown promising results regarding the use of herbal remedies for cancer prevention and treatment, demonstrating the potential of natural sources of chemotherapeutic agents (Table 1) (Huang et al., 2021; Ali et al., 2023).

**TABLE 1:** Marine-derived drugs approved for clinical trials (Nastrucci et al., 2012; Dyshlovoy and Honecker, 2020; Singh, 2022).

	Drug	Drug class	Source (marine organism)	Mechanism of action
1	Brentuximab vedotin (Adcetris™)	Antineoplastics	Sea hare *Dollabellaauricularia*/cyanobacteria	Blocks the step between G2 to M phase to initiate tumor apoptosis
2	Cytarabine, Ara-C (Cytosar-U, Depocyt^®^)	Antineoplastics	Sponge *Cryptotheca crypta*	It has antiviral and immunosuppressive functions
3	Eribulin mesylate (Halaven^®^)	Macrolide	Sponge *Halichondriaokadai*	Prevent outer growth of cytoskeleton via cell cycle prohibition
4	Trabectedin (Yondelis^®^)	Alkaloid	Caribbean tunicate *Ecteinascidia turbinate*	Prohibits DNA repair, replication, and transcription. Interlinks with DNA Small groove
5	Enfortumabvedotin	Antineoplastics	Cyanobacterium *Caldorapenicillata*	Cause apoptosis by cell cycle inhibition via interrupting with microtubular network

**FIGURE 4:**
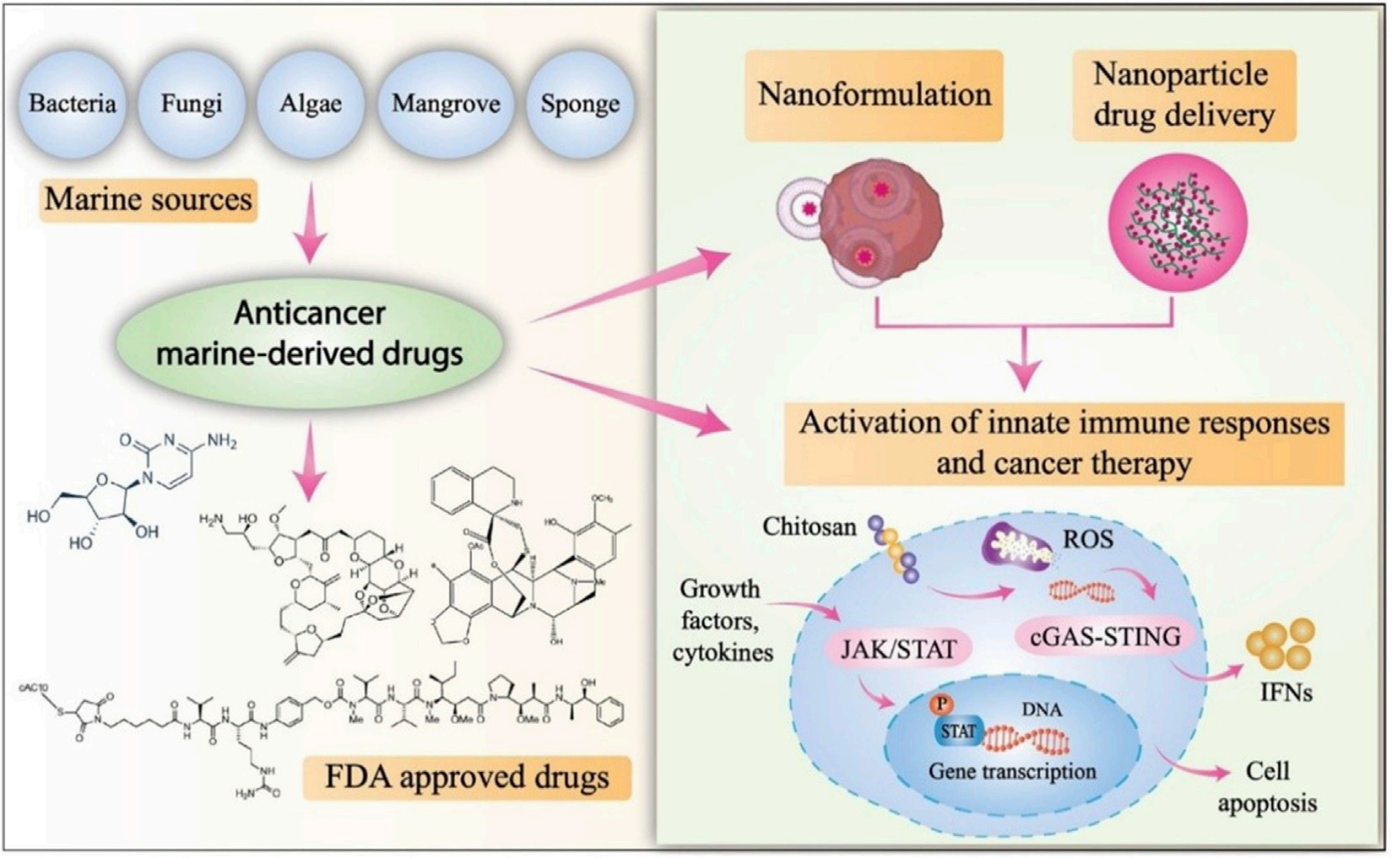
Regulation of JAK/STAT signaling pathways by Marine-based FDA-approved anti-cancer drugs.

The marine environment is a great source of NPs derived from marine sources, which could be used to develop anticancer drugs in the future. Advancements in anticancer drugs have resulted in an upsurge in clinical validation and isolation of different chemotherapeutic treatments for cancer. However, owing to the challenges and limitations discussed here, only a few FDA-approved anticancer drugs derived from marine sources are currently available (Butler et al., 2014). The use of chitosan in the development of drugs derived from marine organisms has the potential to be lucrative in the delivery of anticancer drugs. The cGAS-STING-mediated immune signaling pathway is critical; however, it has received little attention in anticancer therapy and requires further research. In addition, various JAK/STAT signaling pathways, such as immunity, cell death, and tumor formation, are regulated by a small number of anticancer mediators (Figure 4) (Saeed et al., 2021).

## Genomics and proteomics for mining natural products (NPs) in plant and marine sources

Additional core factors for current drug development focused on NPs include improvements in expertise in NP biosynthesis and the design of methods for analyzing and handling genomes. In the genomes of producer species, two main features facilitate the identification of the biosynthetic genes. Several NPs are polyketide- or peptide-based and contain enzymes expressed by numerous genes with closely preserved modules, such as polyketide-synthase synthase (PKSs) and non-ribosomal peptide synthetases (NRPSs) (Ziemert et al., 2016).

**FIGURE 5:**
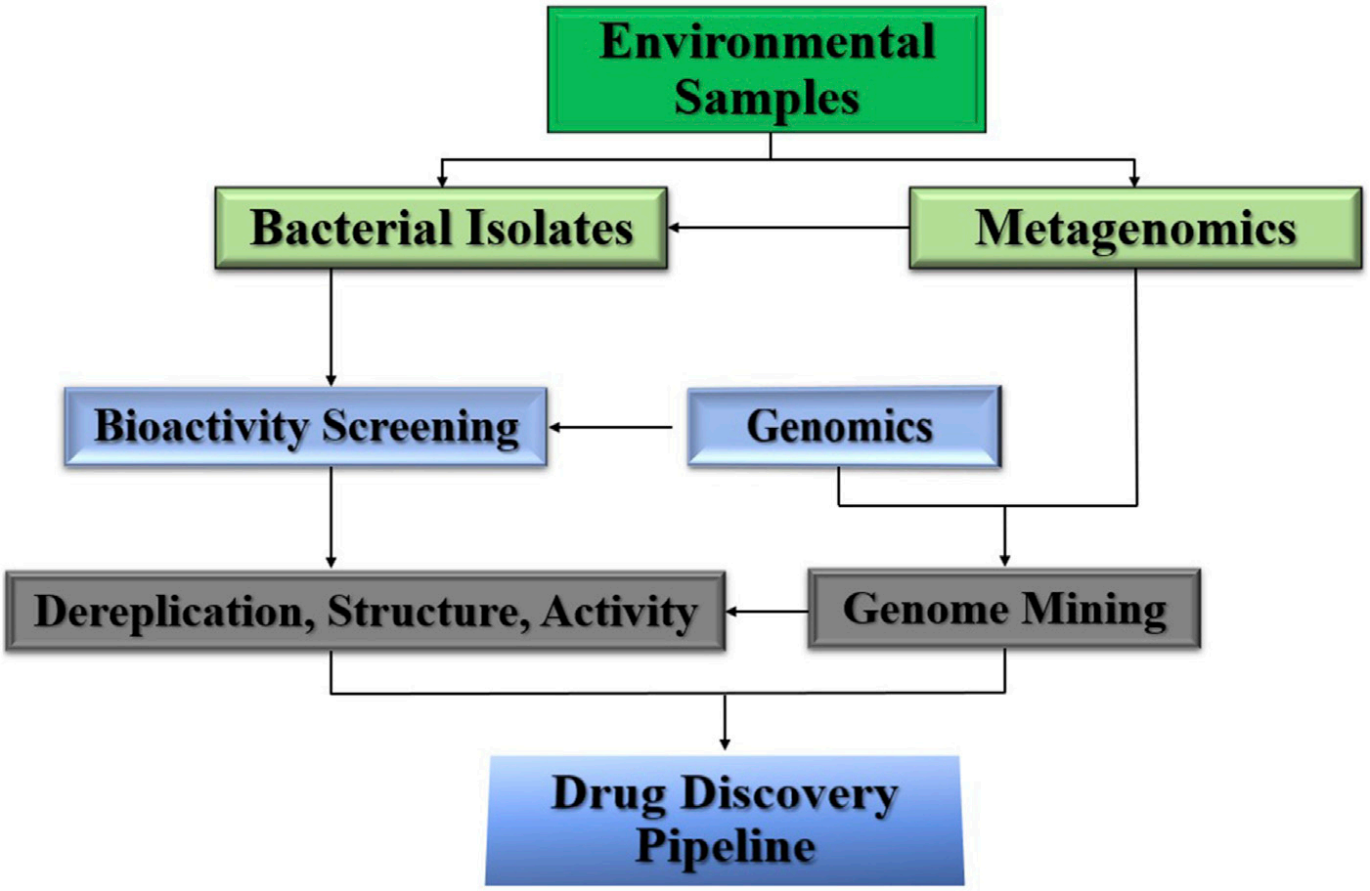
Drug Discovery via genome mining.

Genome mining focuses on gene surveys that can be used to identify NP biosynthetic gene clusters for the biosynthesis of scaffold structures. Prioritizing gene clusters for further work is possible through advancements in biosynthesis and computational bioinformatics methods, indicating whether the metabolism of clusters has fresh or existing chemical scaffolds, thereby encouraging dereplication (Weber and Kim, 2016). Such predictive methods can be used to accelerate the detection of NPs and to assess metabolic substance stereochemistry in conjunction with spectroscopic techniques. In addition, computer methods such as Bi G-SCAPE (the sequence similitude study of biosynthetic gene clusters) and CORASON (a phylogenetic technique used to reveal historical linkages) have been created to expand genomic mining from a single genome to a full generation of microbiomes or strain collections. Drug discovery via genome mining is shown in Figure 5 (Navarro-Muñoz et al., 2020).

The identification of new NPs can also be supported by phylogenetic analyses of the well-identified communities of promising phytochemical sources. A recent analysis associating bioactive compound profiles and phylogenetic details in myxobacteria has revealed a relationship between taxonomic gaps and secondary metabolite families. Bioactive compound profiles have also been linked directly to the phylogenetics of filamentous fungi. These species are abundant in bioactive compounds, as shown by LC-MS experiments under controlled circumstances of their metabolite conditions (Dalinova et al., 2020; Talha et al., 2022). Synchronous genomic and phylogenomic studies have indicated that specific gene clusters involved in secondary metabolite biosynthesis play unexplained roles, even in the genomes of well-studied classes of organisms. In addition to the study of the lack of identified resistance determinants, the phylogenies of biosynthesis gene clusters have recently been a priority for representatives of the antibiotic glycopeptide family representatives may have new activities. This resulted in the discovery of inhibition of peptidoglycan remodelling by the recognized antibiotic and the newly discovered compound, carbomycin (Culp et al., 2020).

Many microorganisms cannot be cultivated, or genetic modification techniques are not adequately designed, making it difficult to access their NP production capacity. However, in organizations that are well-charged and simpler to cultivate and genetically modify, biosynthetic NP gene clusters may be cloned and heterologous. The objective of this study was to improve the availability of plum compounds in heterologous hosts compared to that in wild strains (Sucipto et al., 2017). Vectors with large DNA inserts are required for cloning the entire NP biosynthetic gene cluster. Development was performed on cosmids with 30–40 kb inserts, fosmids with 40–50 kb bacteria, and artificial bacterial chromosomes. Self-replicating fungal chromosomes (FCCs) with inserts >100 kb were formed in the fungal gene groups. FACs were used to create a versatile tool, FAC-MS, which allowed for the classification of fungal biosynthetic gene groups and their respective NPs to an unparalleled extent. FAC-MS resulted in the discovery of 15 new bioactive compounds, including a novel macro-lactone, valactamide A, by screening 55 biosynthetic gene clusters in various fungal organisms (Clevenger et al., 2017).

Many biosynthetic gene clusters may not be expressed under traditional crop conditions, even in cultivable microorganisms, and they may be a significant source of drug-like NPs.Various methods for identifying specific NPs need to be used, such as sequencing, bioinformatics studies, and heterologous expression of silent biosynthetic gene clusters. Experimental NP scaffolds from cultivable strains have already been developed (Mao et al., 2018). The latest antibiotic, taromycin A, was also detected through direct cloning and heterologous expression when a biosynthetic NRPS 67 kb cluster was transferred from S. Coelicolor to S.*C. sp*. CNQ-490. A platform focused on transformation-related recombination cloning (TAR) was created to migrate the biosynthetic gene clusters. BACs are an alternative for the heterologous expression of large clusters of biosynthesized genes, which can directly clone and handle the large biosynthesis of *S. cerevisiae*, and maintain and manipulate the *E. coli* vector to heterologously express clusters of cloned genes in *Actinomyces* after chromosomal incorporation (Yamanaka et al., 2014). The need to clone and manipulate broad genomic regions inhabited by biosynthetic gene clusters and the inability to find an appropriate host that offers all the conditions required to manufacture subsequent NPs have heterologous manifestations. Through selective genetic manipulation, biosynthetic gene clusters may be triggered directly inside indigenous microorganisms, usually requiring the addition of regulatory activation components or removal of inhibitors, such as oppressors or binding sites (Sidda et al., 2014).

The CRISPR–Cas9 innovation is a different way to swap regulatory elements. The potential of this method has been demonstrated in recent studies, showing that CRISPR-Cas9 insertion into promoters can effectively trigger various biosynthetic complexes in several *Streptomyces* species, contributing to the development of unique metabolites, including novel polyketides (Zhang et al., 2017). CRISPR–Cas9 has often been used to synthesize diverse and previously unknown varieties of antibiotics, including amicetine, thiolactomycin, phenanthroviridine, and 5-chloro-3-formylindole, which are encoded by two well-known, mostly re-discovered actinomycete strains (Culp et al., 2019).

**FIGURE 6:**
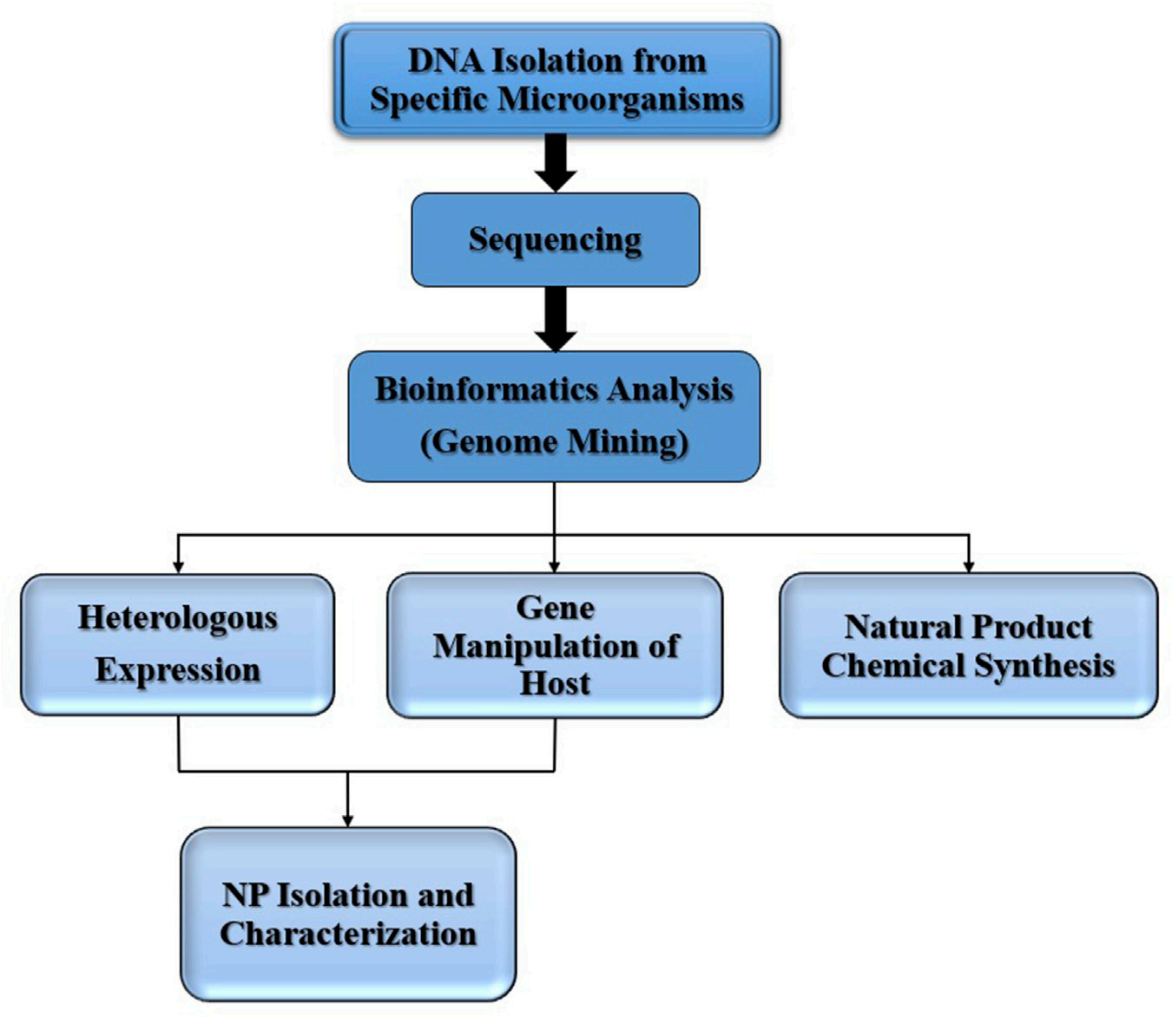
Natural products discovery by genome mining.

Sequence-based strategies, bioinformatics, and heterogeneous expression may also allow for the development of new NPs from bacterial strains that have not yet been cultivated (Figure 6). Scientists have researched, for instance, lipopeptides with calcium-binding patterns in metagenomes of 2,000 soil samples for biosynthetic gene clusters. Malacidins were detected by heterologous expression of a 72 kb desert land-based biosynthetic gene cluster in a *Streptomyces* albus host strain (Hover et al., 2018). They are members of a calcium-dependent antibiotic group (CHAP). However, this metagenome-based exploration strategy is more appropriate than the discovery of entirely different groups in addition to the aforementioned methods (Chu et al., 2016). The arrangement of the gene clusters in the human microbiome is expected to be accompanied by chemical synthesis. One of the strengths of this ground-breaking method is the separate cultivation of the microbial and heterologous genes.

Certain bioactive compounds originally obtained from aquatic animals may be symbiotic materials, and genome mining will help to characterize these NPs. For example, bioactive sponge components *Theonellaswinhoei* were developed by bacterial symbiotics, with the discovery of gene clusters for misakinolide and thionamide biosynthesis characterizedsingle-celled genomics of *Candidatus Entotheonellaserta*. ET-743 (trabectedin), originally extracted from the *Turbinate Ecteinascidia tunicate*, is another example of a marine NP derived from a bacterial symbiont. A meta-analysis has shown that the bacterial symbiont Candidatus Endoecteinascidia frumentensis produces clinically utilized contraceptives (Rath et al., 2011). Certain bioactive compounds originally obtained from aquatic animals may be symbiotic materials, and genome mining will help to characterize these NPs. For example, bioactive sponge components Theonellaswinhoei were developed by bacterial symbiotics, with the discovery of gene clusters for misakinolide and thionamide biosynthesis characterization by single-celled genomics of Candidatus Entotheonellaserta. ET-743 (trabectedin), initially extracted from the Turbinate Ecteinascidia tunicate, seems to be another illustration of marine NP derived from a bacterial symbiont. A meta-analysis showed that the bacterial symbiont Candidatus Endoecteinascidia frumentensis produces clinically utilized contraceptives (Rath et al., 2011).

Certain bioactive compounds originally obtained from aquatic animals may be symbiotic materials, and genome mining will help characterize these NPs. For example, bioactive sponge components Theonellaswinhoei were developed by bacterial symbiotics, with the discovery of gene clusters for misakinolide and thionamide biosynthesis characterization by single-celled genomics of Candidatus Entotheonellaserta. ET-743 (trabectedin), originally extracted from the Turbinate Ecteinascidia tunicate, a marine NP derived from bacterial symbiont. A meta-analysis demonstrated that harvests of the bacterial symbiont Candidatus Endoecteinascidia frumentensis can be clinically utilized as a contraceptive (Rath et al., 2011).

Plant-based microbiomes often include a vast repository for discovery, originally extracted from plants, and were later found to produce microbial endophytes of newer bioactive NAP-hydrates (e.g., maytansine, paclitaxel, and camptothecin antitumor agents). A recent study by Helfrich et al., which detected hundreds of new biosynthetic gene cluster genomes from 224 *Arabidopsis thaliana* blades, is illustrative. To choose one particular species for more genomic analyses, a mixture of bioactivity screening and mass imagery spectrometry has contributed to the isolation of a trans-Acyltransferase PKS-driven NP with an unparalleled structure (Helfrich et al., 2018).

Selective NP biosynthetic genetic engineering may be high if the productive organism is difficult to grow, or if the output of an NP is insufficient for complete NP characterization. The synthesis of vioprolides, a depsipeptide class of anticancer NPs, and the antifungal Cystobacterium violaceus Cb VI35 by many orders of magnitude have been enhanced by reasonable and heterological genetic engineering. Furthermore, this approach produces non-natural vioprolide analogs (Yan et al., 2018). Correspondingly, the development of promoter engineering of biosynthetic gene clusters, along with heterologous expression, has resulted in a 7-fold increase in cytotoxic NP decorasol output and 328 times the rise in spinosad output, an insecticidal macrolide of Saccharopolyspora spinosa (Song et al., 2019).

New progress in biosynthetic engineering has made it possible for the development of analog NPs to be quicker and more effective, including the development of rapid techniques and recombination of PK S gene cluster modules, NRPSs, and assembly lines of NRPS–PKS and clarifying the mechanisms for the release of polyketide chains that contribute to the structural diversification of NPs (Kosol et al., 2019; Masschelein et al., 2019). Examples of biological engineering that extends to many significant NPs include immunosuppressant rapamycin analogs, mithramycin, bleomycin antitumor agents, and nystatin antifungal agents (Brautaset et al., 2008).

## Discovery assays for NPs from sources

**FIGURE 7:**
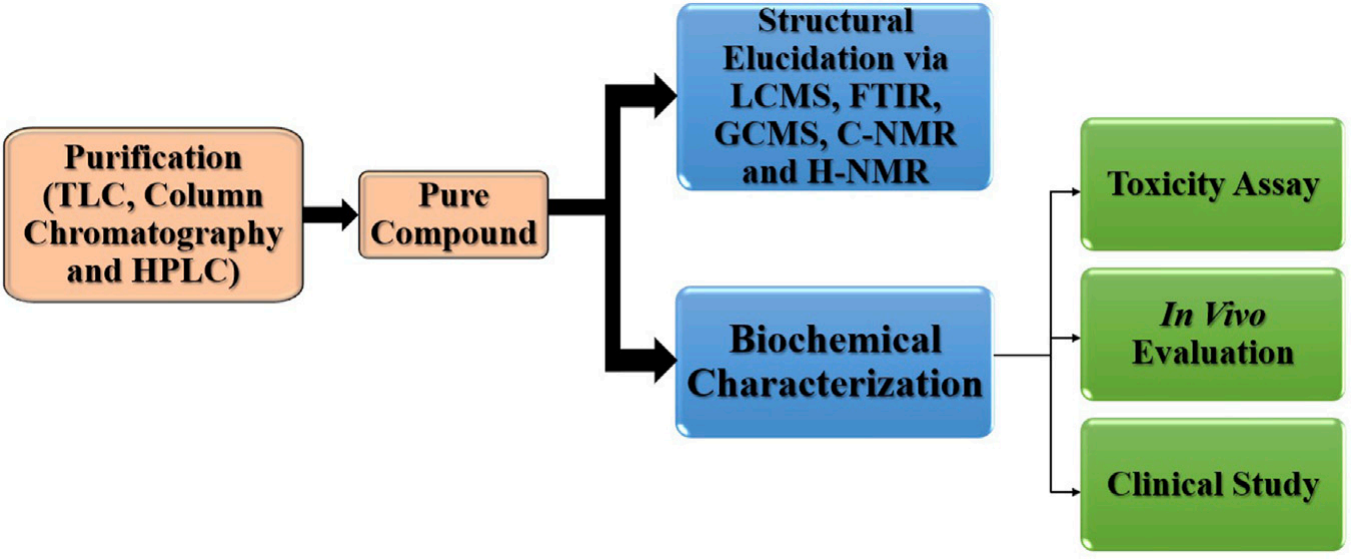
Steps of drug purification for clinical trials.

Chemical, biological, or physical testing must be performed to determine the objective compound (s) of a complex natural product extract. Currently, the study of NPs is based more on the separation of active compounds (assay-oriented isolation) than on the isolation of extracted molecules. The desired molecules may possess chemical groups, physical properties, or biological activity. Therefore, extraction and separation procedures should provide appropriate tests. Figure 7 shows the entire process from drug extraction and purification to clinical administration (Simm et al., 2018).

### Selection of an appropriate assay

When conducting tests on NPs, the following key aspects should be considered. (1) To exclude all insoluble content, samples dissolved or suspended in a solution other than the initial extraction solvent must be filtered or centrifuged. (2) To avoid interference with the test, the acidic or basic samples were re-adjusted to the initial pH. (3) All assays included positive and negative controls. (4) To assess where the bulk of target compounds exists, the test should ideally be at least semi-quantitative, or samples should be assayed in a sequence of dilutions. The test must be sufficiently adaptive to identify low-concentration active components (Sarker et al., 2006).

### Physical examinations

Physical tests can include comparisons of the different chromatographic and spectroscopic activities of the target compound with established standards, such as HPLC, TLC, LC-MS, and CE-MS LC-NMR. Chemical experiments include many chemical studies to classify drug characteristics. For example, FeCl_3_ can be used to determine the phenolic content, Dragendorff’s Alkaloid Reagent, and DPPH for antioxidant compounds (Sarker and Nahar, 2012).

### Biological assays

Bioassays play pivotal roles in drug discovery and development. These tests were used to study the composition of the crude extract, chromatographic proportion, combination, or pure substance using biological methods (e.g., antibacterial, antifungal, anticancer, anti-HIV, and antidiabetic). *Bioassays can include in vivo* (clinical studies and animal experimentation), *ex vivo* (isolated tissues and organs), and *in vitro* (e.g., cultured cells) systems. *In vivo* experiments are clinically significant, and may yield toxic results. The costs, vast numbers of research compounds, complicated architecture, patient requirements, and complexity in the manner of action determination are drawbacks of such trials. *In vitro* bioassays, which are optimal for high-throughput screening (HTS), are faster and require smaller quantities of test substances. However, they may not always accurately reflect *in vivo* results, as outcomes can differ significantly between *in vitro* and *in vivo* settings (Sarker et al., 2006). Currently, the available bioassays are stable, precise, and lower than the picogram quantities of susceptible test compounds. Much can be done in a complete or semi-automated form (e.g., using 96- or 384-well plates).

Several biological tests have been used to evaluate organizational changes, such as testing ecdysteroidal agonists and antagonistic activity (*Drosophila melanogaster* BII cell line experimentation) (Kumarasamy et al., 2002) and serial dilution with resazurin as a cell growth indicator (Drummond and Waigh, 2000). Most modern bioassays focus on microplates and require small amounts of extracts, portions, or compounds for activity assessments (Sarker et al., 2006).

## Techniques for extraction of natural products

NPs must be removed from the biomass before separation and characterization. This method can be used to extract a recognized metabolite, and a formal phytochemical examination can characterize as many metabolites as possible. Typically, only a limited quantity of the substance is extracted from the main extract (Waksmundzka-Hajnos et al., 2004; Shah et al., 2022). This may be carried out by pharmacological analysis of the precise form and quantity of the metabolites in the substance. Once the preliminary extract identifies particular metabolites, isolation of larger amounts can become attractive. This implies gathering more plant content or increasing fermentation levels. Large extractions must be performed in both the cases (Zhang et al., 2021).

### Extraction techniques

Several processes have used chemical and aqueous solvents to produce natural materials.

#### Extraction of natural plant materials

##### Maceration

In this basic, yet commonly used technique, the pulverized plant must be allowed to drink at room temperature in an appropriate solvent in a closed jar. This process was optimal for both original and bulk extractions. The preparation can be increased by occasional or continuous stirring (using mechanical shakers or mixers to ensure homogenous mixing). Extraction ends when a balance is achieved between the metabolite content of the extract and that of the plant substance. The remaining plant substances were isolated from the solution after the processing. This requires a rough explanation of decantation, which is sometimes accompanied by a filtration phase (Sarker et al., 2006). If the powder is too small for filtration, centrifugation may be required. It is normal to perform an initial maceration and then confirm and apply a fresh solvent to the marking to guarantee exhaustive extraction. Both filters were pooled and used regularly. The greatest drawback of maceration is that it takes several hours to several weeks to perform a time-consuming procedure. Comprehensive maceration may also absorb significant amounts of solvents and trigger possible metabolite and/or plant-material degradation. Additionally, these substances are not easily processed if they are poorly soluble at room temperature. However, macerations are less prone to the degradation of thermolabile metabolites when extraction is carried out at room temperature (Ecem Bayram and Gercek, 2019).

##### Ultrasound-assisted solvent extraction

This modified maceration process incorporates ultrasound-assisted extraction. The plant residue was then placed in a vial and ultrasonicated. Ultrasound waves were applied to induce mechanical stress on cells, thereby enhancing the condensation and extraction of compounds from the specimen. Cellular decomposition enhances solvent dissolution and extraction. Recovery performance depends on the intensity, duration, and temperature of the device. Ultrasonication for broad-scale mining is seldom applied; it is often used to remove a limited number of products for the initial extraction. It is widely used to support intracellular substances in plant cells (Oroian et al., 2020).

##### Percolation

Compounds from the plant substances were momentarily immersed in a solvent in a percolator prior to percolation. Further solvent was sprayed over the products and allowed to disperse gradually from the base of the percolator (the drop side). Increased concentrate filtration is not essential because the filter is located at the percolator outlet. Percolation was sufficient for the original and extensive extraction. For maceration, the percolator may be replenished with a fresh solvent to eliminate the plant content and then combine all the substances (Løhre et al., 2017). Percolates can be screened using specific reagents to confirm percolation completion. However, several challenges arise in this process. The depth of the material can influence the extract yield, and finer particles or certain ingredients, such as resins and highly swollen plant components (e.g., mucilages), may obstruct the percolator. Moreover, the solution cannot enter all regions if the liquid is not dispersed homogenously in the bottle (e.g., if it is tightly packaged), and the extraction is incomplete. Both the liquid and solvent temperature interaction periods may also affect the recovery performance (Sarker et al., 2006). Higher temperatures increase the recovery of malleable compounds, but can result in degradation. Another inconvenience of percolation is that large quantities and processes involving solvents are required (Zhang et al., 2018b).

##### Soxhlet

Soxhlet processing is commonly used for the ease of collection from natural products. This process is suitable for both the original and bulk removal. The plant powder was placed on top of the flask under reflux in a cellulose thimble in an extraction chamber. The flask was fitted with a suitable solvent and was heated to reflux. If the thimble accumulated a certain amount of condensed solvent, it was dipped into the bottle below it. This continuous method is a key benefit of Soxhlet extraction (Kumar et al., 2023). The solvent was removed from the flask with fresh solvents and the liquid in the thimble was continually extracted. This reduced the tedious and solvent-consuming extraction of Soxhlets for maceration and percolation. The key drawback of Soxhlet extraction using a solvent’s boiling point because the extract continuously warms up at the boiling point of the used solvent, which can destroy thermolabile compounds and/or cause artifact creation (Alara et al., 2018; Xiao et al., 2020).

##### Pressurized solvent extraction

Pressurized solvent extraction, also known as “accelerated solvent extraction,” is better than most extraction processes and involves extreme pressure to maintain the solvent in a liquid state at high temperatures. This method is ideal for quick and reproducible extraction of various samples (Alvarez-Rivera et al., 2020). The powdered plants were placed in an oven for removal. The solvent was a lant sewereut of the inventory to fill the cell, heated, and pressurized for a certain amount of time at the scheduled temperatures. The cell was flushed with nitrogen oxide and the extract was extracted in a bottle and filtered naturally. Fresh solvents were used to rinse and solubilize the remaining cells. Final purging of the waste using nitrogen gas was achieved. High temperatures and pressures enhance the solvent saturation of the substrate and strengthen the solubility of compounds (Sumampouw et al., 2021). Furthermore, pressurized solvent extraction is less expensive and more environmentally safe than traditional solutions with low solvent specifications. Because the substance is thoroughly dried after extraction, repeated extractions may be performed with the same solvent or subsequent extractions with solvents of increasing polarity. An additional benefit of this technology is that it can be programmed to enhance the productivity. Conversely, for each sample, variables such as the optimum extraction temperature, extraction period, and most appropriate solvent need to be calculated (Zhang et al., 2018a).

##### Supercritical fluid extraction

Supercritical fluid extraction (SFE) requires a supercritical fluid (SF) as the extraction solvent. SF has solvent- and gas-like solubilization and can remove a broad range of NPs. Because of minor pressure and temperature fluctuations, the solvent properties were drastically modified near the critical points. Owing to the extreme attributes of supercritical carbon dioxide (S-CO2), its selectivity, inertness, low cost, antitoxicity, and capacity to remove thermally labile compounds are commonly used in SFE. The polarity of S-low CO2 makes it suitable for nonpolar NPs, such as lipids and volatile oils. S-CO2 can be used as a modifier to significantly improve solvent properties (Gallego et al., 2019).

##### Reflux extraction and steam distillation

During reflux extraction, the plants were soaked in a solution attached to a condenser in a circular bottom bowl. The solvent was heated before reaching the boiling point. The solution was then recycled in a flask as the vapor was diluted. Steam distillation is a related procedure that is widely used to remove essential oils from plants (a complex mixture of volatile constituents). Plants were coated (dried or fresh) in a bottle attached to a condenser. Upon heating, the condensation and distillation (separated into two invasive layers) of the vapors (composition of the essential oil and water) were deposited in a graded condenser-connected tube. The aqueous stage was recirculated in a bottle and the volatile oil was stored separately. The optimal conditions for extraction must be calculated (e.g., distillation rate) based on the quality of the substance to be removed. The key drawback of reflux extraction and distillation is the risk of degradation of the thermolabile materials (Sarker et al., 2006).

##### Decoction

A considerable quantity of water-soluble impurities were present in the decoction extract. However, this decoction method cannot remove thermolabile or explosive material. Ginsenosides are hydrolytic, dehydrated, decarboxylated, and added during decoctions (Zhang H. et al., 2018). They are typically added to the decoction process during later stages to ensure maximum extraction of their beneficial compounds.

##### Marine natural product extraction

Because of the wide variety of microbial universes, the selection, identification, and cultivation of pure strains that may contain bioactive metabolites is challenging. The analysis of microbial metabolites usually begins with the processing of soil samples, because several microorganisms are present on the ground. To identify new strains, a broad range of habitats should be investigated (Jiménez, 2018). Typically, the obtained sample is formulated as a brine solution. Appropriate dilutions of the supernatants were prepared on a stable matrix (agar). Selective media (e.g., the Gram-negative medium MacConkey), antibacterial/antifungal applications (e.g., nystatin for the inhibition of mold and fungal growth), and/or specific requirements for incubation (e.g., incubation of thermophilic strains at 40C) are generally used to isolate other species (Liang et al., 2019). Each colony was collected and subcultivated on various media several times until it was clean (morphologically and microscopically). Pure strains are typically preserved in the presence of a cryoprotective agent in liquid nitrogen or by freeze-drying (Zhao et al., 2019).

The isolated strains were transferred from the reserve to liquid broth to allow for cell growth and metabolite development. Cultivation (or fermentation) was initially performed in fluid medium flasks before the strain was transferred to small fermenters (certified stainless-steel vessels). The entire procedure was scaled up, and the culture was cultivated in a bottle of nutrients that was sealed in a rotating shaker at a given temperature (Sarhan et al., 2019). This allows for reasonably good control of biomass growth and metabolite processing. Initial studies have also been conducted to optimize the cultivation conditions and increase metabolite production. The culture was cultivated under stable conditions while conducting studies in a small fermenter, which could be scaled down until the effects of other critical metabolite manufacturing parameters (e.g., aeration, stirring speed, temperature, pH, oxygen, and carbonic acid concentration) were refined and external pollution was not identified. To ensure replicable efficiency, growth of the producer strain is necessary, which might not be the case because crop morphologies are different from flakes (for example, actinomycetes and filamentous fungi may develop two different morphologies, hyphae or pellets), although they grow in fermenters (Sarker et al., 2006; Shams ul Hassan et al., 2021).

### Isolation techniques

#### Classical or older chromatographic techniques

##### Thin-layer chromatography (TLC)

Efficient visualization and detection are important for pure compounds in both the analysis and preparation phases of TLC, and inadequate detection can lead to low recovery of substances from the sorbent. The identification of these compounds is normally non-destructive (ultraviolet). The only drawback of UV detection is that non-absorbing UV compounds at 254 or 366 nm are invisible and spray detection is essential. However, the main benefit of UV detection is that it is nondestructive, and compound detection can be achieved quickly by separating UV lamps from providers such as CAMAG, which are commonly commercially accessible. Please do not use these lamps to directly contact the eyes or skin, because UV light is mutagenic. Spray detection depends on the color reaction of the compound on the TLC platform with the fine brush of a spray canister introduced on the platform (Sicker et al., 2019).

##### Preparative thin-layer chromatography (PTLC)

PTLC has long been a common isolation tool mainly because of its universal access to natural product chemistry researchers, graduates, and students. The popularity of high-pressure liquid chromatography (HPLC) and countercurrent chromatography (CCC) has decreased in the recent years. However, in contrast to these techniques, PTLC does not require expensive instrumentation. Separations can be performed quickly, and the insulation volume usually falls within 1–1 g intervals, which is adequate for explaining the structure. The basic steps of PTLC are described in this section, with emphasis on the preparation, operating panels, and advantages and drawbacks of PTLC (Kassim et al., 2019).

##### Open-column chromatography (OPC)

Column chromatography is used to isolate a single chemical compound from a mixture. Chromatography can be used to distinguish substances from adsorbents through differential adsorption. Compounds pass through the column at varying speeds such that they can be separated into fractions, which is widespread because a broad variety of solvents can be used for several adsorbents (standard, reversed, or otherwise). This procedure is suitable for scales ranging from micrograms to kilograms. The key advantage of column chromatography is that the stationary step of the method is comparatively low cost and disposable, which avoids cross-contamination and stationary recycling phase deterioration. This technique can be used to transfer the solvent by gravity or by utilizing a compressed gas to drive the solvent through a column (Zhang H. et al., 2018).

##### Flash chromatography (FC)

FC is primarily used to quickly break crude extracts or grossly purify fractures. The mobile stage was flushed through a closely closed glass column or pre-packed cartridges, using nitrogen or compressed air via the stationary phase. A smaller particle size (∼40 mm in the case of silica) should be used, thus increasing the peak resolution compared to open-column chromatography. Online peak sensing, which is normally performed by connecting the sensor to a UV sensor, is feasible. Supercritical liquid chromatography (HPLC) and FC are exciting new options, but require considerably higher equipment costs. Examples of successive FC applications are also observed. TLC separations for stationary stages have been proposed (Baldé et al., 2021) for the FC method production.

#### Modern chromatographic techniques

##### High-performance thin-layer chromatography (HPTLC)

Thin-layer chromatography analysis is one of the main identity checks in most pharmacopeia manuscripts. Classic organizations (CO) utilize pharmaceutical guidelines as a framework for achieving QC standards and existing good industrial practices (cGMPs). TLC is a durable, easy, fast, and effective method for quantitative analysis of high-performance thin-layer chromatography (HPTLC). HPTLC is a TLC-focused analytical technology designed to improve component precision and quantitative component evaluation. Some improvements, such as the use of higher-quality TLC plates in the standard process, have enabled the improved resolution of finer particles. By repeatedly developing the board and utilizing multiple production kits, the separation may be further enhanced. As a result, HPTLC provides a higher resolution and lower detection limits (LODs) (Attimarad et al., 2011).

HPTLC is one of the most widely used analytical systems in pharmaceutical, clinical, investigative, biochemical, cosmetological, food and beverage, environmental, and other fields. For instance, it is the only chromatographic approach that allows the findings to be shown as a picture owing to its various advantages. Other advantages include simplicity, low price, simultaneous sample review, high sample capability, fast findings, and numerous detection opportunities (Amina et al., 2018).

##### Vacuum liquid chromatography (VLC)

Unlike other chromatography column forced-flow techniques, VLC does not require pressure but a vacuum to accelerate the amount below, thereby accelerating the fractionation process. VLC column beds are normally made of 40–60 mm silica or reversed silica. For the sample (liquid, inactivated silica, or diatomaceous earth) and mobile stages, which are always gradient by step with increasing elution force, the open end of the column is readily available (e.g., hexane to methanol for silica columns). Owing to its usability and large sample capacity, VLC is a common tool for the fractionation of raw extracts. The eluted fractions were evaluated for their structures using TLC. The Sticher1 analysis demonstrated the use of VLC in various compound groups, including sterols, flavonoids, alkaloids, triterpene saponins, or coumarins (Maurya et al., 2018).

#### Chromatogram

Chromatograms have been developed by the researchers of the Compendium of Organic Synthesis Methods as a prepared, centrifugally accelerated, angular, thinly layered chromatography. It can substitute for TLC plates, tiny columns, and HPLC. The total size is 30 × 35 × 30 cm. The isolated sample was added to the middle of a rotating disc covered with a thin sorbent layer. The elution of different elements, along with the solvent, by the solvent created spherical bands spun off from the rotor tip. This new storage device carries a single output tube for the eluate (Elissawy et al., 2019).

##### Solid-phase extraction

Solid-phase extraction (SPE) is an extractive procedure whereby compounds dissolved or suspended inside a liquid mixture, according to their physical and chemical properties, are isolated from other compounds in the mixture. Analytical laboratories concentrate and purify the samples for analysis using a solid-phase extraction system. Massive phases can be used to separate analytes of interest in a broad range of matrices, including vomit, skin, water, drinks, soil, and animal tissue (Capriotti et al., 2019).

##### Droplet counter-current chromatography (DCCC)

DCCC is dependent on the splitting of solutes into a steady stream of mobile phase droplets and surrounding fixed-phase column. Balancing an appropriate solvent mixture results in the formation of a pair of immiscible phases. Depending on the separation problem, either the light or heavy phase may be chosen as the mobile phase. The instrument, shaped by 200–500 vertical columns, is filled with stationary, heavier phases, whereas the lightweight phase is in the ascending mode, which is bound to the capillaries by the TFT series. Separation was performed by delivering a mobile step to the bottom of the first column. Droplets that went up to the top of the column were created, and Teflon pipes were delivered to the bottom of the next column, thus producing fresh droplets. Only the mobile phase flowed under the appropriate conditions. At the end of the column series, the mobile process with the mixture components was performed using a fraction collector sorted according to their partition coefficients (Nahar and Sarker, 2020).

##### High-performance liquid chromatography (HPLC)

The column used for the processing and purification of the NP was octadecyl silica (RP-18). However, some laboratories are beneficial for the provision of high-quality HPLC columns with different modern generations in the following phases: cyano, phenyl, triazole, secondary and tertiary amines, β-cyclodextrin, and dihydroxy propane for efficient NP isolation and purification. The isolation of NPs has been widely utilized. Some of these may be used in the HILIC mode. Approximately 20 years ago, the term “hydrophilic chromatography association” (HILIC) 171 was introduced. HILIC can be considered a new method of partition chromatography among chromatographic principles and standard and inversion-phase chromatography. The stationary stage of the HILIC column was polar, in the form of silanol, amino, or loaded groups. Mobile processes can include high concentrations of organic solvents (typically, acetonitrile) and pH may be used for selectivity (Kabiri et al., 2017).

##### Hyphenated techniques (e.g., HPLC-PDA, LC-MS, LC-NMR, LC-MS-NMR)

Chromatographic and spectral approaches were combined using hyphenated technologies to account for both. Chromatography contains a combination of pure and almost-pure chemical elements. Spectroscopy provides selective recognition details using the normal spectrum of the repositories (Nagajyothi et al., 2017).

## Plant and marine NPs in precision medicine

NPs are tiny substances that can be prokaryotes or eukaryotes derived from living beings. Several therapeutically valued NPs have been recognized and introduced as current medicines, or are currently being tested as possible new drugs (Waltenberger et al., 2016; Tewari et al., 2018). Precision medicine is a medical model focuses on providing patients with personalized healthcare. In recent years, many success stories across the world have begun, along with the Precision Medicine Initiative introduced by the United States National Institutes of Health (NIH) and the Chinese Precision Medicine Initiative recently launched by the Chinese Government (Cyranoski, 2016; Zhang H. et al., 2018), which will contribute to defining the future of medical treatment through the exponential advancement of research fields such as genetics, genomics, and systems biology. A rapid website search using the keywords ‘natural product’ or ‘targeted therapy’ or ‘precision medicine’ revealed a steady (natural product) growth in clinical interest in these scientific fields to be relatively fast.

NPs are used for the modulation of nuclear receptors, anti-inflammatory effects, lipid metabolism, regulation of microRNAs and other non-coding RNAs, treatment of malaria, autophagy, osteoporosis, cancer, and aging. (Atanasov et al., 2018).

## Advantages of natural products (NPs) based drugs over synthetic drugs

NP studies offer valuable insights into new strategies for drug development. Their diverse chemical structures derived from their natural origins make them relevant for current drug research. Their compact structural organization and natural bioactive compounds give them an advantage over synthetic compounds (Namadina et al., 2020). Strategies for the success of NPs include chemistry, biochemistry, pharmacology, and molecular modelling. Their immense scaffold (structural and chemical) diversity complements synthetic compounds, with 45% of best-selling medicines originating from NPs (Lahlou, 2013). NPs often have higher molecular weights and fewer hydrogen, halogen, or sulfur atoms but more oxygen atoms. NPs exhibit complexity with more tetrahedral carbon atoms, rings, and chiral carbon centers (Lahlou, 2013). In addition, NPs serve as a valuable source of novel medicinal products and are strong lead compounds for drug development. In drug discovery, a vast proportion of NPs is derived from various mechanisms and complex carbon skeletons. Although secondary metabolites originate from natural sources in living systems, they have also been recognized for their enhanced pharmacological properties compared to those of synthetic molecules (Lahlou, 2013).

### Drugs available in market-based on plant and marine NPs

#### Anti-inflammatory

Inflammation is the first biochemical reaction to an infection or irritation of the immune system. However, an increasing number of serological studies have shown a substantial chance of developing multiple human diseases, such as acute zealous inflammation or recurrent inflammatory reactions. Thus, it is necessary to avoid or alleviate disorders such as organ transplants, allergies, and autoimmune diseases by managing or altering inflammation.

In the last few decades of natural products, considerable efforts have been made worldwide to explore new formulations of natural products with greater effectiveness and lower toxicity for thousands of years in the treatment and prevention of human diseases around the globe. In reality, it is remarkable that most immunocompromising drugs originally come from natural products such as mycophenolic acid (MPA), cyclosporine A (CsA), rapamycin, tacrolimus (FK506), and fingolimod (FTY720) (Yuan et al., 2006; Xie et al., 2019; Shams ul Hassan et al., 2021).

#### Antibiotic

Historically, natural products have been crucial in the discovery and synthesis of antibacterial agents. In recent years, interest in such systems has decreased, but the rapid growth of resistant bacterial strains has forced re-evaluation to identify novel chemical skeletons with antibacterial activities for drug development (Moloney, 2016).

The use of natural products and their derivatives has become an excellent approach to the treatment of various infectious diseases, particularly in the fight against drug-resistant microbial strains. In particular, the presence of new architectures and pathways of action in these natural products can guide the development of new chemotherapies. Three compounds with novel modes of action, walkmycin B, waldiomycin B, and signamycin B, were isolated from actinomycete metabolites as inhalers of histidine kinase. New antibiotics for TB, tuberlactomicin A, and caprazamicines have been identified, and amycolamicin has been described as an antibiotic against *Staphylococcus aureus*, which is immune to anti-methicillin (Hassan et al., 2017; Igarashi, 2019).

#### Antiparasitic

Higher plants and microscopic species are used as renewable sources for the discovery of modern medicinal products. A few other natural products with varied molecular structures have shown anti-parasitic power and provide interesting guidelines for the synthesis of current and desperately required anti-parasitic agents in the laboratory. Among other aspects, the battle for novel anti-parasitic agents must consider the following problems. Currently, only a few medications are available in the market to cure several common parasitic diseases. Target precision can increase drug efficiency, allowing greater use of the biochemical and biological properties of each parasite. In addition, certain clinical medications, including natural products such as quinine and emetine, have variable potencies and adverse consequences, and require long-term implementation. Testing natural materials offers an opportunity to discover new molecules with a particular structure that can be further improved by half or entirely synthetic processes with high action and selectivity (Kayser et al., 2003).

#### Antifungal

Although antifungal agents are at different stages of clinical development with new mechanisms of action, their number is comparatively limited compared to that of other therapeutic indications. An appropriate preclinical conduit pathway is required, and the processing of natural products contributes significantly to this. Natural products are often well validated to be biological, with several instances accepted in their natural form as therapeutics or semi-synthetic derivatives (Newman and Cragg, 2016b). Actinomycetes are the most abundant source of natural bacterial materials, which is also the case for antifungal plants. There were also three isolated examples of non-actinomycetes and two of algae. Elephant Dung *Streptomyces* albolongus strain YIM 101047 developed several bafilomycins during laboratory fermentation. Marine sponges are a valuable source of new natural products and more than 10 indications of antifungal activity have been identified. The aqueous extract of the Plakortishalichondroides-Xestospongia deweerdtae symbiotic two-sponge relationship provided a series of natural peroxide products. Over the last decade, approximately ten antifungal traces have been reported in plants. The flavonoid (e)-6-(2-carboxyethenyl) apigenin was extracted from the extract of the Brazilian medicinal plant Mimosa caesalpiniifolia Benth, generally referred to as ‘sabiá' or ‘sansão-do-campo. This compound inhibited *C. krusei,* with an IC50 of 44 nM, whereas C. glabrata was inactive. Isoflavonoid vatacarpan with a 1 μg/mL MIC against C. albicans was extracted from the roots of Vatairea macrocarpa (Benth.) using bioassay-directed fractionation (Aldholmi et al., 2019).

#### Cardiovascular

Cardiovascular health is a critical concern and dietary supplements play an important role in improving wellbeing. Scientific, epidemiological, and clinical evidence suggests that dietary supplements have a cardioprotective effect on primary and secondary cardiac disease control. Different cardioprotections have been recorded for the management and cure of cardiovascular disorders, such as flavonoids (cellar, pulse, red wine, tea, and cocoa), and olive oil (The Mediterranean diet, rich in olive oil, has been associated with reduced cardiovascular risk. Olive oil contains monounsaturated fats that support heart health.), Omega 3 (omega 3) fatty acids (fish oils and fish products), and lycopene (abundant in tomatoes, lycopene is a powerful antioxidant. Regular consumption has been linked to a lower risk of heart disease), resveratrol (found in grapes and red wine), and resveratrol have gained attention for their potential cardiovascular benefits. It may improve blood flow and protect against heart disease), coffee (moderate coffee consumption has been associated with a reduced risk of heart disease), soy (soy-based products containing plant compounds called isoflavones, which may contribute to heart health), garlic, dark chocolate, magnesium, and berries (Shukla et al., 2010; Tf et al., 2023).

#### Anesthetics

Many commonly used drugs come from natural sources, particularly plants. Drugs and plants are directly linked to the use of herbal drugs or plant ethnomedicines. The descriptive medicinal products of plant-based involve antimalarial quinine (Cinchona officinalis, Rubiaceae), cardiotonic digitoxin (Digitalis purpureae, Plantaginaceae), antitussive codeine (DPS), and analgesic salicylate (PS) and its derived aspirin. Medicinal herbs are reservoirs of crude medicines and reservoirs of crude medicines and bioactive compounds, which could contribute to new pharmaceutical structures. Since traditional times, medicinal plants and herbs have served to relieve pain due to illness, accidents, and surgery, and some have led to the development of modern anesthesia. Cocaine, the first localized anesthetic with a particular drug alkaloid, exhibits a partial composition and pharmacological activity with many plant terpenoids. The anesthetic addition of morphine and the injectable d-tubocurarine muscle relaxant were obtained from the opium poppy and vine plant curare of the arrow toxin (Tsuchiya, 2017).

#### Anticancer

More than 60 percent of today’s anticancer medicines are extracted from natural sources in one manner or another. Nature is indeed an ample source of bioactive and diverse chemotypes, and although some of the real isolated natural products have developed into efficacious drugs themselves, such molecules also help to predict the production of more efficient analogs and prodrugs using chemical methodologies, such as whole or combination. Advancements in composition may also contribute to a more efficient administration of drugs to patients or to the production of efficient chemotherapeutic agents by conjugating toxic natural molecules with monoclonal antibodies and multiple carriers specifically attacking tumor epitopes of concern. The importance of natural products in identifying and evolving new anticancer agents and the significance of interdisciplinary cooperation in optimizing current biological routes from naturally occurring products have been widely studied (Cragg and Pezzuto, 2016).

#### Diabetic

Natural additives have long been used in conventional diabetes medication. Nopal (prickly pear cactus), fenugreek, karela, gymnema, ginseng, tronadora, chromium, and alpha-lipoic acid are the most popular products that varies from person to person. Nopal is the most widely used natural hypoglycemic agent of Mexican origin. Karela is used most often by people from Asian countries. All of these agents also earned a universal appeal. Studies have demonstrated single or multiple modes of action for a selected range of goods. A high soluble fiber content contributes to many of these factors.

Based on available information, several commonly used natural products may reduce blood glucose levels in patients with diabetes. Common natural products often have a long-standing history of conventional use, and pharmacists who are more familiar with these products are better able to advise patients about their proper use (Shapiro and Gong, 2002).

#### ADHD

The most frequent brain development disorder in infants is attention-deficit/hyperactivity disorder (ADHD), which is a neurodevelopmental disorder with core symptoms of hyperactivity, carelessness, and impulsivity. In addition, although this disorder is diagnosed quite often during childhood, it can also affect a person’s life. In view of their academic, social, and family implications and the harm of comorbidities and subsequent substance abuse, it is vital to develop better therapies for ADHD (Hinshaw et al., 2015; Hassan S. S. U. et al., 2022). Given the possible adverse effects and effectiveness of current pharmacological ADHD procedures, there has been increasing interest in developing experimental therapies, such as natural product-oriented ADHD treatments, including natural plant or herbal medicinal products, vitamins, minerals, and amino acids. These conventional medicines endear parents who want more “natural” procedures for their children. Approximately 50% of children with ADHD use these therapies, either alone or in combination with other medicines or substances. NPs consequent ADHD management comprising corresponding ADHD intercessions, such as vitamins, minerals, and other dietary supplements, report discoveries of scientific trials that assessed the effectiveness and protection of these interventions and the probable mechanism(s) by which these mediators progress ADHD symptoms (Ahn et al., 2016).

## Conclusions and future standpoints

Advancements in technology and computational science are expected to shape the future of drug discovery, leading to more efficient and cost-effective processes. To achieve sustainable drug discovery, identifying and addressing the root causes of unsustainable healthcare practices is crucial. This involves analyzing the entire drug development process, from the initial research stages to production and distribution, to pinpoint areas where inefficiencies and environmental harm occur. Implementing targeted strategies to mitigate these issues is essential for minimizing environmental impacts while meeting medical needs.

A significant aspect of sustainable drug discovery is the use of eco-friendly practices and innovative technologies. Biomarkers and bioinformatics have become invaluable tools in drug discovery, enabling the identification of specific molecular targets and development of more targeted and efficient therapies. By utilizing biomarkers and bioinformatics, the drug discovery process can be streamlined, reducing unnecessary experiments and minimizing resource consumption.

Another key aspect is the integration of societal needs and ethical considerations into R&D processes through responsible research and innovation (RRI). This approach fosters collaboration, transparency, and accountability in the healthcare industry, ensuring that sustainability and environmental impacts are considered.

Additionally, the healthcare industry must adopt sustainable practices and harness innovative technologies to minimize environmental impacts. This includes reducing energy consumption through energy-efficient technologies and practices, implementing proper waste management systems, and exploring alternative sources of pharmaceutical products. The depletion of natural resources and release of harmful substances into the environment are significant ecological consequences of drug discovery that must be addressed.

Furthermore, the application of artificial intelligence (AI) and machine learning (ML) in drug discovery can significantly expedite the process by identifying potential drug-disease relationships that would otherwise remain undetected. AI-powered platforms can be used to analyze vast datasets, leading to more accurate predictions of drug effectiveness and safety profiles. AI and ML will also be used to model complex biological systems and predict how potential drugs will interact with these systems.

Plant and marine base natural products are currently mostly preferred bioactive sources for new drug discovery compared to terrestrial plants and microbes, which are non-marine due to owing diversity in their chemical structure and showing tremendous biological and pharmacological activities. Nonetheless, the use of plant and marine-derived natural products to acquire sufficient material to sustain a complete production of various drugs effort is dependent on the different techniques for sample collecting, separating, and enhancing structural characterization by various spectrometric methods for a biologically active molecule, due to having diverse functions in the treatment of various fatal diseases. Plant and marine-derived drugs play a very important role in the treatment of cancer, coronary artery disease, viral, bacterial, fungal, and inflammatory diseases.

This review employs different techniques for the isolation and characterization of and for NP drugs ability such as TLC, PTLC, OPC, FC, HPTLC, VLC, DCC, HPLC, and hyphenated techniques such as the HPLC-PDA, LC-MS, LC-NMR, and LC-MS-NMR with recently advance in their applications. In addition to this, various protocols for sample preparation, tools for data processing, and many other applications in natural products-based drug discovery are outlined, highlighting their limitations and advantages. The main challenge is the extreme level of complexity and structure diversity present in natural organisms. To know about the genetic combination and biosynthesis of NP genomics and proteomics is also discussed which is helpful in the biosynthetic development of the new drugs and all the plant and marine-derived natural products, opening new doors for the treatment of various harnessing diseases. Moreover, various extraction techniques have limited sensitivity, efficiency, selectivity, scalability, and environmental impact and often need complex matrices, solvent-intensive, time consuming and costly resulting in less affordable and viable. These challenges can be overcome by using biodegradable reagents, better-quality materials like nano-sorbents, and eco-friendly solvent-free techniques. Machine based learning might improve specificity and sensitivity, while automation and microfluidics reduces the size efficiently and offers more affordable studies. By using these efforts future looks very safe and tangible benefits with new natural products with improved biological and pharmacological activities. Thus, present review assists the development of new plant and marine-based natural products in drug discovery and provides an indication of hope for improved treatment from the vast ocean.
